# Effectiveness of Manual Hysteroscopic Tissue Removal Device for Intrauterine Polyps in Infertile Women in Both Operating and Office Settings

**DOI:** 10.3390/jcm13082244

**Published:** 2024-04-12

**Authors:** Chen Wang, Hui Chen, India Morgan, Valeriya Prytkova, Belinda Kohl-Thomas, J. Preston Parry, Steven R. Lindheim

**Affiliations:** 1 Institute of Reproductive and Stem Cell Engineering, NHC Key Laboratory of Human Stem Cell and Reproductive Engineering, School of Basic Medical Sciences, Central South University, Changsha 410008, China; chen_wang2019@hotmail.com; 2 Clinical Research Center for Reproduction and Genetics of Hunan Province, Reproductive and Genetic Hospital of CITIC-XIANGYA, Changsha 410221, China; chh1966421@163.com; 3 Texas A&M School of Medicine, Temple, TX 77807, USA; india.weinkauf@bswhealth.org (I.M.); belinda.kohlthomas@bswhealth.org (B.K.-T.); 4 College of Medicine, University of Central Florida, Orlando, FL 32827, USA; vprytkova@ucf.edu; 5 Department of Obstetrics & Gynecology, Baylor Scott & White Health, Temple, TX 76508, USA; 6 University Mississippi Medical Center, Jackson, MS 39216, USA; jpreston@parryscope.com; 7 Health Sciences Shreveport, Louisiana State University, Shreveport, LA 71115, USA

**Keywords:** hysteroscopic polypectomy, morcellation, endometrial polyps, operative hysteroscopy, office hysteroscopy, mechanical tissue resection

## Abstract

**Background**: Mechanical hysteroscopic tissue removal (mHTR) systems are widely used for removing intrauterine pathology. Given the startup and procedural costs for electrically powered mechanical units, disposable manual mHTR systems have been developed. **Methods**: With little published, we describe its effectiveness for hysteroscopic intrauterine polypectomy. **Results**: One-hundred fifty-seven infertile women underwent hysteroscopic polypectomy with the manual mHTR device. Complete removal was accomplished in all but three cases, with blood loss being <10 mL and all specimens deemed sufficient for histopathologic diagnosis. **Conclusions**: These results suggest that the disposable manual mHTR system is effective in removing endometrial polyps. Head-to-head comparisons with other alternative technologies are needed.

## 1. Introduction

Endometrial polyps (EPs) are focal intrauterine lesions composed of sessile or pedunculated endometrial glands, stroma, blood vessels, and fibrous tissues. Both isolated and multiple polyps can be found in the uterus and vary in size from a few millimeters to several centimeters [1]. EPs may be asymptomatic but may present as abnormal uterine bleeding and/or infertility [1]. With respect to the latter, the prevalence of EPs for infertile women before in vitro fertilization and embryo transfer (IVF-ET) has been reported to range between 6% and 32% [2,3,4].

Some evidence suggests that EPs have an adverse effect on fertility, and there is an association between hysteroscopic polypectomy and increased rates of natural pregnancy [2]. The possible mechanisms related to infertility include adverse effects on endometrial thickness, local endometrial blood supply, uterine cavity shape, and receptivity, suggesting a molecular mechanism in reduced pregnancy rates in women with EPs [2]. As such, the surgical removal of EPs is recommended for infertility patients, with evidence suggesting improved success for natural conception and IVF [5].

Hysteroscopy with resectoscopes and traditional mechanical resection with scissors and/or graspers remains standard for the evaluation and treatment of the pathology of the cervical canal and endometrial cavity [6,7]. However, the use of scissors and/or graspers often necessitates the insertion and removal of the hysteroscope and/or the instruments through the working channel several times to ensure the complete removal of pathology. While more suitable for isolated polyps, this can increase the operative time and difficulty for more extensive diseases.

In 2005, Emanuel et al. first reported on the electromechanical hysteroscopic tissue morcellator (mHTR), which combines a disposable mechanical cutting device using a rotating tube inside a cutting window that simultaneously resects and aspirates the polypoid tissue into a collection bag. This eliminates the need for frequent insertion to remove the tissue [8]. mHTR systems are driven mechanically by an electrically powered control unit and have advantages in that they do not use electrocoagulation and use a physiologic saline solution as distension and irrigation media instead of non-physiologic, electrolyte-free solutions used in monopolar high-frequency resectoscopy. Previous studies have demonstrated a significant reduction in operating times for the removal of polyps and myomas when using mHTR systems compared to the resectoscope, with associated low rates of adverse events, high physician acceptance, and significant health-related, quality-of-life improvements up to twelve months following resection [9,10]. Further, while some have reported that hysteroscopic mHTR for benign intrauterine lesions have no adverse effects on the implantation rate, clinical pregnancy rate, miscarriage rate, and live birth rate [11], others have reported significantly higher pregnancy rates (65.1% vs. 54.6%, *p* = 0.045) and that time to pregnancy was shorter (13.1 ± 7.9 months vs. 16.3 ± 8.2 months, *p* = 0.04) compared to electroresection. They concluded that in addition to effectively removing intrauterine lesions, mHTR devices may facilitate the healing of the endometrium, thereby shortening the postoperative time to pregnancy [11,12]. Increasingly, electromechanical mHTR devices have become widespread for removing intrauterine pathology in both the operating room and office setting.

Newer lower cost manual mHTR devices have come on the market with previous studies confirming their efficacy in hysteroscopic polypectomy [13,14,15]. Nonetheless, head-to-head comparisons with the resectoscope, cold-knife resection, and elecromechanical mHTR devices are limited. This includes a recent report that compared the electromechanical mHTR to the Resectr™ 3 mm (Minerva Surgical, Inc., Sanat Clara, CA, USA) manual mHTR, and overall, it was non-inferior to the electromechanical mHTR (TruClear™, Medtronic, Minneapolis, MN, USA) for hysteroscopic polypectomy when comparing procedure duration, conversion rates, and incomplete resection rates, including a 10% reduction in average instrument setup time and a 30% increase in average removal time. However, self-perceived surgeon safety, efficacy, and comfort scores favored electromechanical morcellation [16].

Recently, a newer manual mHTR, The Polygon Resection Device™, (Polygon Medical Devices, Holliston, MA, USA, and distributed as PolyGone^®^ OriGyn Medical, Hangzhou, China) has become available. We report our collective experience and the effectiveness of this low-cost manual mHTR in both the operating room and office setting.

## 2. Materials and Methods

This retrospective study was approved by the University of Central Florida Institutional Review Board (IRB 20230726) and the ethics committee (IRB LL-SC-2023-034) of Reproductive and Genetic Hospital of CITIC-XIANGYA (Hunan, China) and conducted in compliance with privacy act guidelines.

### 2.1. Study Population

**Figure 1:**
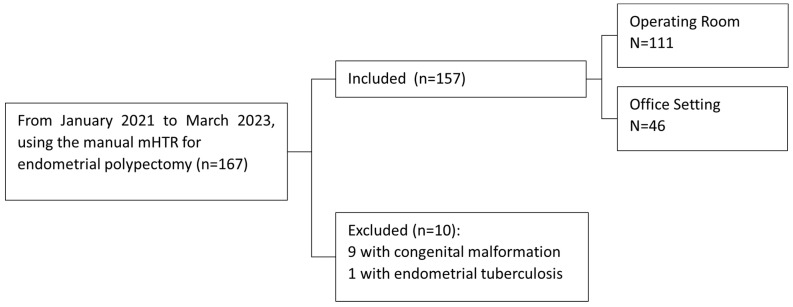
Flow diagram of cycles included the analysis.

From January 2021 to March 2023, all records from 167 consecutive women with infertility planning to undergo IVF-ET who had identified EP pathology by either a transvaginal ultrasound, sonohysterography, or hysterosalpingogram were reviewed. During this time period, all patients underwent hysteroscopic polypectomy using the manual mHTR at the physician’s discretion with traditional mechanical resection with scissors-polyp graspers or a resectoscope as a back-up in the event of treatment failure. Patients with congenital malformations (*n* = 9) and endometrial tuberculosis (*n* = 1) were excluded. The analysis included 157 infertile women who underwent manual mHTR resection during the study period with 111 performed in the operating room setting and 46 in the outpatient office setting with at least one year of postoperative follow-up data, as depicted in Figure 1. The histopathologic diagnosis of endometrial polyp at hysteroscopy was required for inclusion.

### 2.2. Surgical Procedure

All procedures performed from the Chinese-based program were performed in the operating room suite as the routine standard of care which included monitored anesthesia care (MAC). The USA-based program offered either the operating or office setting provided that participants had no history of significant chronic diseases, history of anesthetic or surgical complications, and uterine filling defects that were estimated to be less than 2 cm in size and were given pre-procedure NSAIDs and allowed to choose between no anesthesia and a paracervical block. All women in the Chinese and USA groups were either placed on oral contraceptives prior to their procedure or had their procedure performed early in the follicular phase with a screening urine ß-hcg.

**Figure 2:**
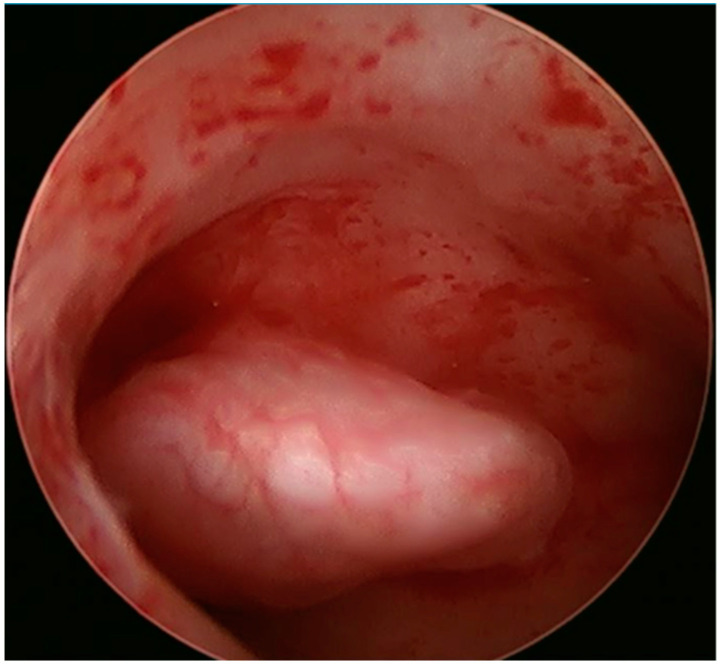
Visual inspection identified a 1.5 cm polyp on the right lateral wall of the uterus.

Procedures performed by the Chinese group utilized a 6.2 mm 12° OriScope hysteroscope with a 9Fr working channel (OriGyn Medical, Hangzhou, China), while the USA program utilized either the 6.2 mm 0^0^ lens hysteroscope with a 3.3 mm working channel (MyoSure Hysteroscopic Omni^®^ 4K System, Marlborough, MA, USA) or a disposable 5.5 mm 12^0^ lens with a 3.3 mm working channel (AcuVue™, Los Altos, CA, USA) to initially identify and confirm the location, size, and relationship of endometrial polyps to the surrounding tissues (Figure 2). Normal saline was used as the distension medium administered manually by elevating the fluid bag three to four feet above the uterus with pressures not exceeding 100 mm Hg.

### 2.3. Manual Hysteroscopic Tissue Removal System (mHTR)

**Figure 3:**
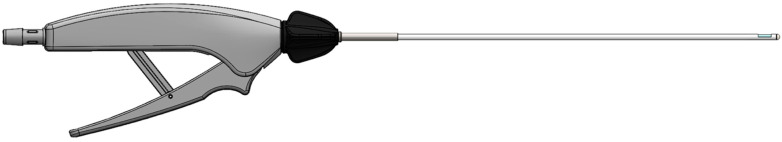
Polygon mHTR device.

**Figure 4:**
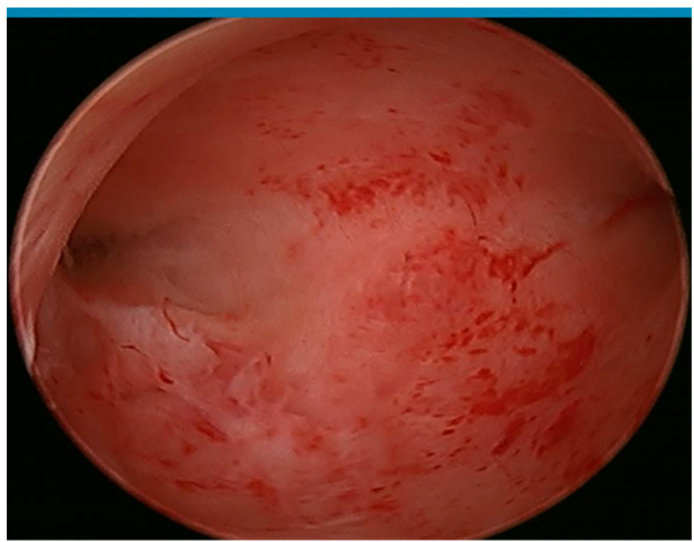
Manual mHTR resection which took approximately 15 device activations and 90 s.

After the diagnostic component of the procedure was performed, the same mHTR device was used by both groups but marketed with different tradenames (Polygon, Polygon Medical Devices, Holliston, MA, USA/PolyGone™ OriGyn Medical, Hangzhou, China). In particular, the manual mHTR device has a shaft device that is designed to fit into the working channel of any hysteroscope that includes a 3.0 mm (Fr) straight working channel. Polygon/PolyGone consists of a hand-held trigger assembly, a handle with a built-in mechanical gear drive assembly, a manually rotating torsional wheel, a cutting assembly, and a suction assembly. The device is activated by the physician squeezing the trigger as there are no electrical or powered connections necessary for operation. When the trigger is released, the elastic force is extended and transmitted to the cutting edge through a gear drive, achieving a single straight and vertical cutting of the tissue in an instant. A rotation knob enables the physician to rotate the cutting bay into an orientation that aligns with the desired specimen. The barb fitting provides an attachment port for a vacuum system, which pulls the specimen into the cutting window where it is resected and aspirated from the uterus (Figure 3). The opening is aligned with the polyp site under direct hysteroscopic vision, and the tissue is suctioned through the suction tube and collected in the suction vial until no obvious polyp remains under hysteroscopic view (Figure 4). Upon completion, the resected pathology in the suction bottle is filtered and sent for pathologic evaluation.

### 2.4. Outcome Measured

While we detail comparisons for each setting, the primary objective was to describe the clinical application of the Polygon/PolyGone device with an emphasis on complete resection rate and adequate histopathologic diagnosis. A complete polypectomy rate is defined as the detachment and retrieval of all visible polyp tissue (single or multiple polyps) such that no polyp remnants remained within the uterine cavity under hysteroscopic vision. A complete resection rate is defined as the number of patients with Polygon/PolyGone complete resection/total number of patients ×100% [17].

### 2.5. Statistical Methods

Categorical variables were described as frequencies and percentages, while continuous variables were expressed as mean ± standard deviation (SD). Comparisons of operating room-based and office-based polypectomy procedures were compared with chi-squared or Fisher’s exact test between categorical variables and with *t*-test for comparisons of continuous variables using the SPSS statistical package 21.0 (SPSS Inc., Chicago, IL, USA). While hysteroscopic procedures are increasingly being moved into the office setting with minimal or no anesthesia for reimbursement and time efficiency benefits, the standard still remains the operating room for many countries, including China, where MAC or general anesthesia is routinely provided.

## 3. Results

One-hundred fifty-seven cases were reviewed and detailed in the analysis, including 111 performed in the operating room and 46 in the office setting. The demographic characteristics are described in Table 1. The mean age of the women was 32.9 ± 5.5 yrs, ranging between 28 and 38 yrs. Cases performed in the operating room setting had a significantly lower BMI than those in the office setting (22.1 ± 3 vs. 23.9 ± 4.5 kg/m^2^, *p* < 0.01).

**Figure 5:**
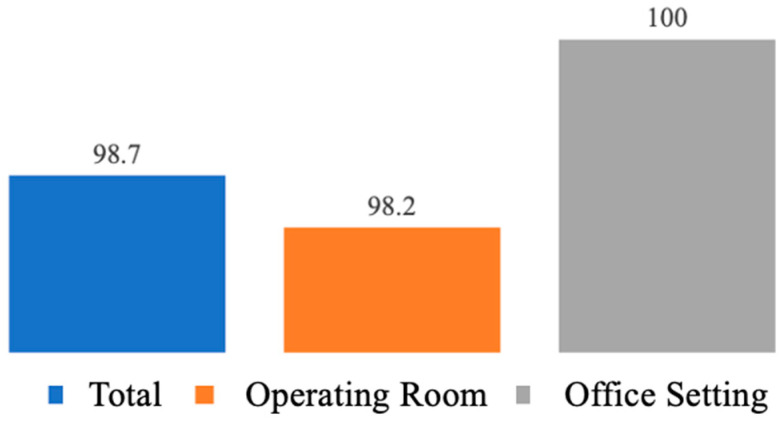
Percentage of cases with complete removal of endometrial polyps.

MAC anesthesia was administered for all operating room cases, while in the office setting, 10.8% (5) had a paracervical block, and 89.2% (41) had a vaginoscopic approach not requiring any anesthesia. All polyps were 2.0 cm or smaller with one (21.7%, (34)); two (9.6%, (15)); three (5.1% (8)); four (5.1%, (8)); and five or more polyps (58.9%, (92)), respectively, identified. Those cases performed in the operating room had a significantly greater number of polyps than office procedures (three or more, 93.7% vs. 8.7%, *p* < 0.001). Complete resection rate was accomplished in 98.1% (154) of cases. Two cases in the operating room required graspers due to the cornual location of the pathology and a fibrous band that necessitated scissors for complete resection (Figure 5).

As expected, the procedural time of operating room cases (27.1 ± 6.4 min, range 15–60 min) was significantly longer than office setting cases (7.3 ± 1.8 min, range 5–10). Blood loss was <10 mL for all cases, and all specimens had adequate and benign histopathologic diagnosis except for one case with adenomatous hyperplasia (Table 2). No intra- and post-operative complications were noted in either group.

## 4. Discussion

In our descriptive report, we detail the ease and effectiveness of the Polygon/PolyGone manual mHTR device in the removal of intrauterine polyps both in the operating room and office setting. Complete resection was accomplished in 98.1% of cases with sufficient tissue for histopathologic diagnosis. This suggests that disposable, hand-held manual mHTR systems can be effective in removing endometrial polyps.

The efforts to simplify and improve the intrauterine treatment of polyps, myomas, and/or intrauterine adhesions in clinical practice have led to the development of new and smaller devices. These include the electromechanical HTR systems, including the TruClear™ (Medtronic), MyoSure^®^ (Hologic Inc., Marlborough, MA, USA), and Intrauterine BIGATTI Shaver^®^ (Karl Storz, Tuttlingen, Germany), which have helped facilitate the removal of intrauterine pathology. Previous studies comparing the resectoscope and mHTR have shown advantages in the application of mHTR. According to the FDA’s MAUDE (Manufacturer and User Device Experience) database, mHTR reduces life-threatening complications, including fluid overload, uterine perforation, and bleeding, compared to hysteroscopic resectoscope [18,19,20]. Additionally, in a recent meta-analysis of 498 patients from five studies, mHTR systems have been reported to significantly reduce surgical time for polypectomy and submucosal myomectomy compared to conventional resection [21]. Moreover, a network meta-analysis of eight randomized controlled studies showed that mHTR had the highest likelihood of optimal clinical outcomes compared with electroresection with respect to operative times, success rates, and complications [22].

However, while electrically powered mHTR devices offer significant advantages, including efficiency, precision, lower complication rates, and short learning curves [12,13], they are often accompanied by high start-up and operating costs. These include not only the direct costs of purchasing and maintaining electric control equipment but also the indirect costs of installation and commissioning. Together, these factors have limited the widespread use of electromechanical mHTR systems in both the operating room and outpatient settings. In contrast, the manual mHTR devices have provided an alternative with an innovative design concept that significantly reduces these costs while retaining the benefits of electric devices. The single-use equipment design eliminates the need for expensive maintenance and continuous equipment renewal, thereby reducing initial investment costs. Manual mHTR devices also simplify setup and operation procedures, without complicated installation and debugging, making the operation process more convenient. Given the operation process is more intuitive, it reduces the need for training and its associated costs. This makes the manual mHTR an attractive option for the treatment of endometrial lesions.

To our knowledge, there are currently three manual mHTR devices on the market including the MyoSure^®^ MANUAL device (Hologic™), Resectr™ 9Fr (Minerva Surgical), and the Polygon™ (Polygon Medical Devices, Holliston, MA, USA/PolyGone by OriGyn Medical, Hangzhou, China).

van Wessel et al. recently reported on the Resectr™ 3 mm (Minerva Surgical) manual mHTR which was non-inferior to the electromechanical mHTR (TruClear™) for hysteroscopic polypectomy with respect to procedure times, conversion rates, and the incompleteness of resection rates; however, surgeon’s safety, effective, and comfort scores favored the electromechanical morcellator [16]. van Wessel et al. recently reported on the Resectr™ 3 mm (Minerva Surgical) manual mHTR which is used for the removal of endometrial polyps. The cutting window is serrated, and each cutting blade is bi-directional, internally rotates and oscillates, and provides six rotations per handle squeeze. Similar to the Polygon/PolyGone, a handpiece is squeezed which initiates the turning movements of the inner blade, enabling tissue cutting with a controlled suction device to extract resected tissue for pathologic analysis. The MyoSure^®^ MANUAL mHTR is another hand-held device that has been marketed for office use allowing for a quick and convenient removal of tissue, such as fibroids and polyps, with a fully integrated vacuum and detachable trap for pathologic evaluation, which does not require external suction. However, to our knowledge, there are no clinical studies currently reported detailing its efficacy.

In our descriptive report, we detail the ease and effectiveness of the Polygon/PolyGone manual mHTR device for hysteroscopic polypectomy in both the operating room and office setting. Its use is intended to hysteroscopically resect focal lesions including Eps and retained products of conception while providing an efficient procedural time and limited conversion requiring the use of graspers/scissors. We recognize the limitations of our report, including the lack of a comparison group, which limits the generalizability of our results and different surgical approaches by the two surgical centers. We are currently gathering data on long-term issues, including pregnancy outcomes. Nonetheless, further studies are required to (1) establish their long-term efficacy, similar to electromechanical HTR systems for intrauterine polyps and/or myomas; (2) given the Polygon and other manual mHTR systems are not designed to remove submucosal myomas, clinical outcomes regarding their removal as well as retained products of conception are needed; and (3) cost-effectiveness studies are required to assess its full economic benefits.

## 5. Conclusions

Our preliminary findings present a new mHTR system for which the presented data demonstrate the effectiveness of the disposable TR device in the removal of intrauterine polyps in either the operating room or office setting and merits further evaluation to compare it with other current technologies. As patient demand for less invasive approaches to address intrauterine pathology increases, simpler options may accelerate the adoption of manual mHTR procedures for intrauterine pathologies.

## Figures and Tables

**Table 1:** Baseline demographics.

Characteristic	Overall (*n* = 157)	Operating Room (*n* = 111)	Office Setting (*n* = 46)	*p*-Value
Age (yrs)	32.9± 5.5	33.1 ± 4.1	31.4 ± 5.9	0.24
Gravidity	0.9 ± 1.2	0.95 ± 1.24	1.0 ± 1.3	0.92
Parity	0.3 ± 1.2	0.3 ± 0.5	1.0 ± 1.3	0.91
BMI (kg/m^2^) Primary infertility Secondary infertility Total number of polyps	23.0 ± 3.877 (49%) 80 (51%) 6.3 ± 3.0	22.1 ± 3 54 (48.6%) 57 (51.4%) 6.73 ± 2.7	23.9 ± 4.5 23 (50%) 23 (50%) 1.6 ± 1.2	<0.01 <0.01 0.88 <0.001

**Table 2:** Intraoperative outcomes.

Characteristic	Overall (*n* = 157)	Operating Room (*n* = 111)	Office (*n* = 46)	*p*-Value
Anesthesia				<0.001
MAC	111	100% (111)	0	
Paracervical	5	0	10.8% (5)	
None	41	0	89.2% (41)	
Number of polyps				<0.001
1	21.7% (34)	4.5% (5)	63% (29)	
2	9.6% (15)	1.8% (2)	28.2% (13)	
3	5.1% (8)	7.2% (8)	0% (0)	
4	5.1% (8)	7.2% (8)	0% (0)	
5 or more	58.9% (92)	79.3% (88)	8.7% (4)	
Cervical dilation	111 (70.7%)	111 (100%)	0 (0%)	<0.001
Blood loss (cc)	2.6 ± 2.3 (0–10)	2.9 ± 2.3 (1.00–10.00)	0	<0.001
Total time for procedure (min)	26 ± 7.6 (5–60)	27.1 ± 6.4	7.3 ± 1.8	<0.001
Complications	0 (0%)	0 (0%)	0 (0%)	1.0

## Data Availability

The raw data supporting the conclusions of this article will be made available by the authors upon request.
